# The *IL-33*/*ST2* Axis Affects Adipogenesis Through Regulating the *TRAF6*/*RelA* Pathway

**DOI:** 10.3390/ijms252212005

**Published:** 2024-11-08

**Authors:** Shujun Cao, Xuyong Qin, Chengping Li, Lichun Zhang, Shizhong Ren, Wenhao Zhou, Meiman Zhao, Guoli Zhou

**Affiliations:** 1 College of Agriculture and Biology, Liaocheng University, Liaocheng 252000, China; 2210150215@stu.lcu.edu.cn (S.C.); qxy5025@163.com (X.Q.); lichp2008@126.com (C.L.); 2210150219@stu.lcu.edu.cn (S.R.); 2310150224@stu.lcu.edu.cn (W.Z.); 2310150223@stu.lcu.edu.cn (M.Z.); 2 Institute of Animal Biotechnology, Jilin Academy of Agricultural Sciences, Gongzhuling 136100, China; zhang_lich@163.com

**Keywords:** IL-33/ST2 axis, signaling pathways, adipogenesis, TRAF6, RelA

## Abstract

Understanding the regulatory mechanisms of adipogenesis is essential for preventing obesity. Interleukin-33 (IL-33) has recently attracted increasing attention for its role in adipogenesis. The purpose of this study was to explore the function and regulatory mechanism of IL-33 and its receptor suppression of tumorigenicity 2 (ST2) on adipogenesis. Here, Oil Red O staining was used to detect the accumulation of intracellular lipid droplets. Molecular techniques such as qRT-PCR and Western blotting were used to detect the expression of pivotal genes and adipogenic marker genes. Gains and losses of function experiments were used to explore the potential regulatory mechanism of the IL-33/ST2 axis in adipogenesis. Functionally, IL-33 is negatively associated with adipogenesis in 3T3-L1 preadipocytes, while ST2 is positively associated with it, encompassing both the trans-membrane receptor ST2 (ST2L) and the soluble ST2 (sST2). Mechanistically, the IL-33/ST2 axis affects adipogenesis by regulating the expression of the TRAF6/RelA pathway in 3T3-L1 preadipocytes. Downregulating the expression of ST2 suppressed the activation of the IL-33/ST2 axis, which subsequently inhibits the expression of TRAF6. This further attenuates the expression of RelA, ultimately resulting in the suppression of adipogenesis in 3T3-L1 preadipocytes. This study reveals a new mechanism by which the IL-33/ST2 axis regulates the differentiation of preadipocytes and provides a new idea for improving obesity prevention.

## 1. Introduction

Nutritional diet and obesity have always been hot topics of concern [1]. The advancement of the socio-economic landscape and the elevation of living standards have fostered public awareness regarding the importance of physical and mental health, as well as nutritional balance [2,3,4]. Excessive fat intake is prone to causing diseases such as obesity [5,6]. An unhealthy diet leads to the development of obesity, which is characterized by chronic low-grade inflammation and is closely associated with various immune-related disorders [7].

Obesity is a non-communicable disease that can lead to various health complications, such as diabetes [8], hypertension [9], cardiovascular diseases [8,10], and certain types of cancer [11], posing a significant threat to public health. Hence, understanding the mechanism of adipogenesis is crucial for a balanced diet and preventing obesity. In recent years, studies have found that obesity is accompanied by an inflammatory response; the level of TNF-α in the plasma of obese patients and type II diabetes patients is significantly higher [12]. More research is needed to investigate the relationship between certain inflammatory factors and adipogenesis. A deeper understanding of the potential regulatory mechanisms of adipogenesis may become an effective strategy for obesity treatment.

The signaling pathway is a bridge for transmitting information between cells, playing a crucial role in regulating cellular function, disease development, and treatment response. Among them, the IL-33/ST2 axis has been one of the hotspots of attention in recent years. The IL-33/ST2 axis is involved in the signaling processes of various pathways, such as NF-κB, MAPKs, TGF-β, AKT, and mTOR [13], which participate in various biological regulatory processes, such as cell proliferation and migration [14], inflammatory response, and cell differentiation [15]. It is closely related to many diseases, such as asthma [16], cardiovascular disease [8,10], diabetes [8], inflammatory bowel disease, and obesity [10,15,17], and plays an important role in regulating physiological and biochemical reactions in the body. Therefore, we speculate that the IL-33/ST2 axis may regulate the TRAF6/RelA pathway to affect adipogenesis.

IL-33 has been proven to be a “dual functional” cytokine that not only acts as a nuclear inhibitory factor in the nucleus to regulate the expression of target genes but also as an “alarm” secreted outside the cell to bind with ST2L, participating in the regulation of downstream gene expression along the IL-33/ST2 axis [18,19]. Additionally, IL-33 was proven to dampen the transcriptional activity of NF-κB through interacting with the RelA [20]. ST2 produces four variants due to selective promoter and 3′ truncation, among which sST2 and ST2L are the most widely studied [16,21]. Previous study indicates that ST2 selectively expresses more sST2 when IL-33 is overexpressed, competes with ST2L for binding to IL-33, and inhibits the function of IL-33 [13]. ST2L is a receptor complex that binds to IL-1RAcP to form a heterodimer. It acts as a receptor and binds to IL-33, transmitting extracellular signals to the cell and exerting biological functions by activating the expression of multiple signaling pathways [13]. Among them, research has shown that TRAF6 is a signaling molecule shared by the Interleukin-1 receptor (IL-1R)/Toll-like receptor (TLR) family and the TNFR superfamily [22]. It acts as a linker protein to transmit signals from receptor complexes to downstream transcription factor activators and acts as an E3 ubiquitin ligase to modify its own ubiquitination regulation [22,23]. TRAF6 activates the IKK kinase complex through ubiquitination modification [24,25], leading to the activation of MAPK and NF-κB, which release RelA into the nucleus to exert its function [26].

Previous studies have found that the IL-33/ST2 axis regulates perinatal thermogenesis in mice by modulating the expression of UCP1, which affects the differentiation of brown adipocytes [27,28,29]. These results indicate that the IL-33/ST2 axis has a certain biological role in adipogenesis, but its role and regulatory mechanism in the differentiation of 3T3-L1 preadipocytes are unclear. Previous studies focused on IL-33, but the secretion form and regulatory mechanism of IL-33 are currently incomplete. It is indisputable that the ST2L participates in the IL-33/ST2 axis as a receptor. Herein, we investigated the function of the IL-33/ST2 axis in the differentiation of 3T3-L1 preadipocytes. In addition, the signaling pathway involved in IL-33/ST2 was investigated to determine whether it regulates the adipogenesis of 3T3-L1 preadipocytes by regulating the TRAF6/RelA pathway.

## 2. Results

### 2.1. Expression Profile of IL-33/ST2 During Adipogenesis

**Figure 1:**
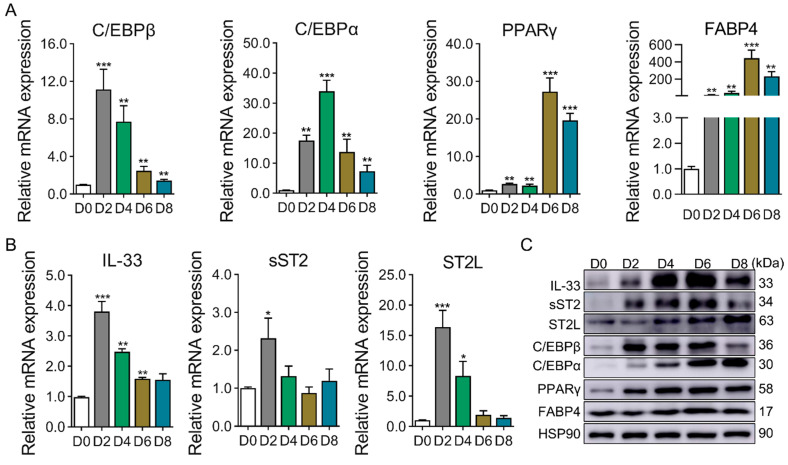
Expression profile of adipogenic marker genes and IL-33, sST2, and ST2L during adipogenesis of 3T3-L1 cells. (**A**) mRNA levels of adipogenic marker genes were detected by qRT-PCR. (**B**) mRNA levels of IL-33, sST2, and ST2L were detected by qRT-PCR. (**C**) Protein levels of IL-33, sST2, ST2L, and adipogenic marker genes were detected by Western blotting. Data were represented as the mean ± SEM (*n* = 3). *, *p* < 0.05; **, *p* < 0.01; ***, *p* < 0.001.

To investigate the expression tendency of IL-33 and ST2 during adipogenesis of 3T3-L1 cells, qRT-PCR and Western blotting were used to detect its expression. With the progress of induced differentiation, the expression levels of adipogenic marker genes (C/EBPβ, C/EBPα, PPARγ, and FABP4) were significantly increased compared with D0 (Figure 1A). The mRNA expression profiles of IL-33, sST2, and ST2L were similar to C/EBPβ at the mRNA level. Their mRNA expression levels demonstrated a tendency for an initial increase followed by a decrease, attaining their peak expression level at D2 of induced differentiation and subsequently declining (Figure 1B). The protein expression levels of IL-33, sST2, and ST2L displayed a progressively ascending tendency along with the induction of differentiation (Figure 1C). Overall, the results suggest that IL-33/ST2 may affect the adipogenesis of 3T3-L1 preadipocytes.

### 2.2. IL-33 Attenuates Adipogenesis in 3T3-L1 Cells

**Figure 2:**
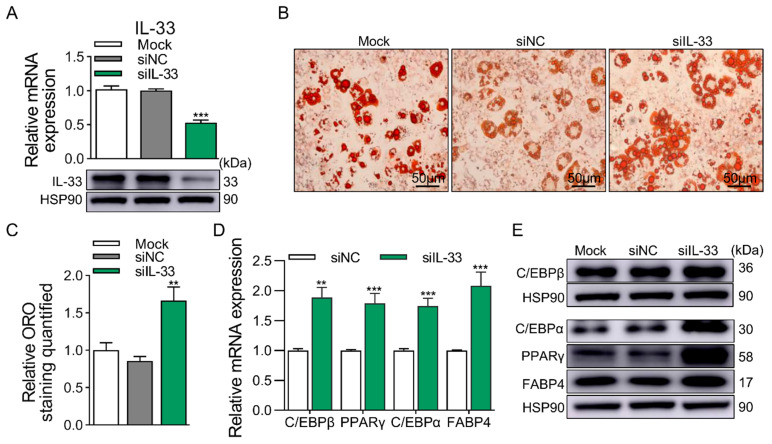
KD of IL-33 promotes adipogenesis of 3T3-L1 cells. (**A**) KD efficiency of IL-33 at mRNA (top panel) and protein (bottom panel) levels. (**B**) ORO staining of differentiated 3T3-L1 cells at D8. (**C**) Quantification of ORO staining. Scale bar, 50 μm. (**D**) The mRNA levels of adipogenic marker genes at D8, except for C/EBPβ at D2, were detected by qRT-PCR. (**E**) The protein levels of adipogenic marker genes at D8, except for C/EBPβ at D2, were detected by Western blotting. Data were represented as the mean ± SEM (*n* = 3). **, *p* < 0.01; ***, *p* < 0.001.

**Figure 3:**
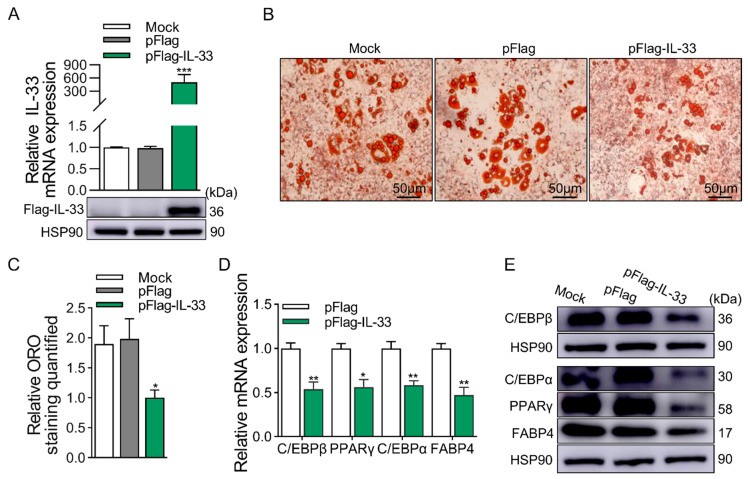
OE of IL-33 attenuates adipogenesis of 3T3-L1 cells. (**A**) OE efficiency of IL-33 at mRNA (top panel) and protein (bottom panel) levels. (**B**) ORO staining of differentiated 3T3-L1 cells at D8. (**C**) Quantification of ORO staining. Scale bar, 50 μm. (**D**) The mRNA levels of adipogenic marker genes at D8, except for C/EBPβ at D2, were detected by qRT-PCR. (**E**) The protein levels of adipogenic marker genes at D8, except for C/EBPβ at D2, were detected by Western blotting. Data were represented as the mean ± SEM (*n* = 3). *, *p* < 0.05; **, *p* < 0.01; ***, *p* < 0.001.

Overexpression (OE) and knockdown (KD) techniques were used to investigate the function of IL-33 on adipogenesis. The mRNA and protein expression efficiencies of IL-33 were detected by qRT-PCR and Western blotting (Figure 2A). KD of IL-33 significantly promoted the accumulation of lipid droplets in 3T3-L1 cells at D8 (Figure 2B), and ORO staining quantified revealed a significant increase in the IL-33 KD group (Figure 2C). The expression of adipogenic marker genes in the cells of the IL-33 KD group was significantly upregulated compared with the control group at the mRNA and protein levels (Figure 2D,E). However, OE of IL-33 produced results that were opposite to those of KD of IL-33. Both the mRNA and protein expression levels of IL-33 significantly increased after transfection with the pFlag-IL33 (Figure 3A). Simultaneously, the IL-33 OE group showed a significant decrease in lipid droplet accumulation and ORO quantification at D8 (Figure 3B,C). Then, the expression of adipogenic marker genes in IL-33 OE group cells was significantly reduced at the mRNA and protein levels (Figure 3D,E). In summary, the above results proved that IL-33 dampens adipogenesis of 3T3-L1 cells.

### 2.3. ST2 Promotes Adipogenesis of 3T3-L1 Cells

**Figure 4:**
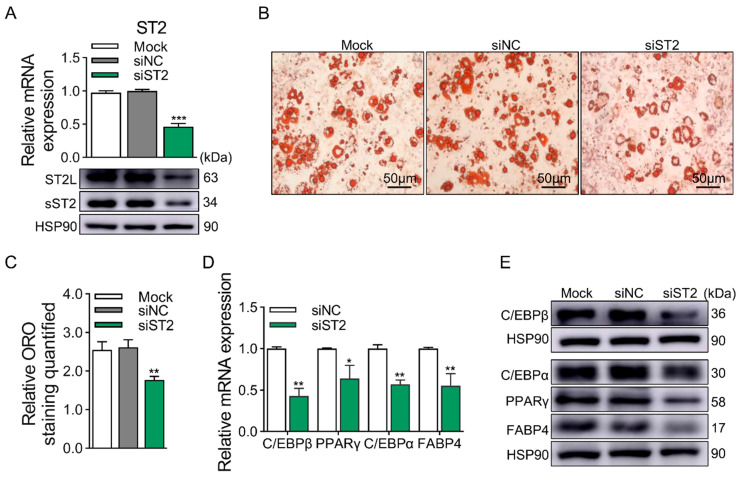
KD of ST2 decreases adipogenesis of 3T3-L1 cells. (**A**) KD efficiency of ST2 at mRNA (top panel) and protein (bottom panel) levels. (**B**) ORO staining of differentiated 3T3-L1 cells at D8. (**C**) Quantification of ORO staining. Scale bar, 50 μm. (**D**) The mRNA levels of adipogenic marker genes at D8, except for C/EBPβ at D2, were detected by qRT-PCR. (**E**) The protein levels of adipogenic marker genes at D8, except for C/EBPβ at D2, were detected by Western blotting. Data were represented as the mean ± SEM (*n* = 3). *, *p* < 0.05; **, *p* < 0.01; ***, *p* < 0.001.

ST2 is mainly composed of sST2 and ST2L, of which ST2L is the receptor for IL-33 and combines with IL-1RAcp to form a receptor complex that binds to IL-33 to exert its biological function. To investigate whether ST2 influences the differentiation of 3T3-L1 cells, we explored the function of ST2 through KD experiments. The siRNA we transfected was designed in the common coding region of sST2 and ST2L, and the expression of sST2 and ST2L was simultaneously downregulated at both the mRNA and protein levels (Figure 4A). The ST2 KD group showed a significant reduction in lipid droplet accumulation and ORO quantification at D8 (Figure 4B,C). Then, the expression of adipogenic marker genes was significantly reduced in the ST2 KD group at the mRNA and protein levels (Figure 4D,E).

**Figure 5:**
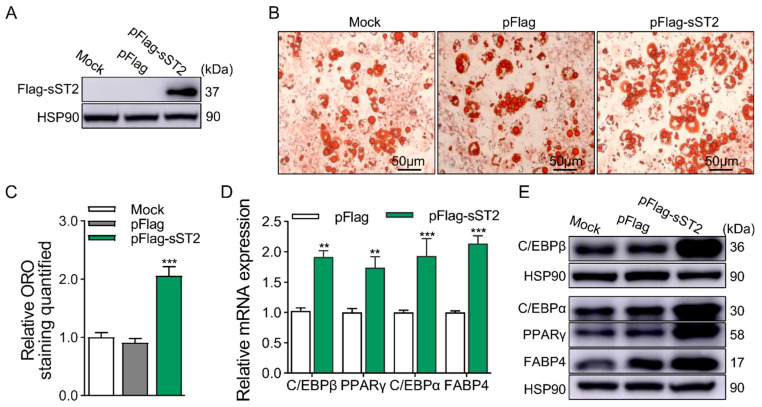
OE of sST2 increases adipogenesis of 3T3-L1 cells. (**A**) OE efficiency of sST2 at protein levels. (**B**) ORO staining of differentiated 3T3-L1 cells at D8. (**C**) Quantification of ORO staining. Scale bar, 50 μm. (**D**) The mRNA levels of adipogenic marker genes at D8, except for C/EBPβ at D2, were detected by qRT-PCR. (**E**) The protein levels of adipogenic marker genes at D8, except for C/EBPβ at D2, were detected by Western blotting. Data were represented as the mean ± SEM (*n* = 3). **, *p* < 0.01; ***, *p* < 0.001.

According to the above experimental results, it seems that we cannot determine the function of the IL-33/ST2 axis in the adipogenesis of preadipocytes. Subsequently, we transfected pFlag-sST2 or specific siRNA of ST2L separately to further investigate the function of sST2 or ST2L in adipogenesis. Flag-sST2 represents the efficiency of sST2 at the protein level (Figure 5A). The results of ORO staining and quantitative analysis showed that the sST2 OE group significantly promoted lipid droplet accumulation in 3T3-L1 cells at D8 (Figure 5B,C). Furthermore, the expression of adipogenic marker genes was significantly increased in the sST2 OE group at both the mRNA and protein levels (Figure 5D,E).

**Figure 6:**
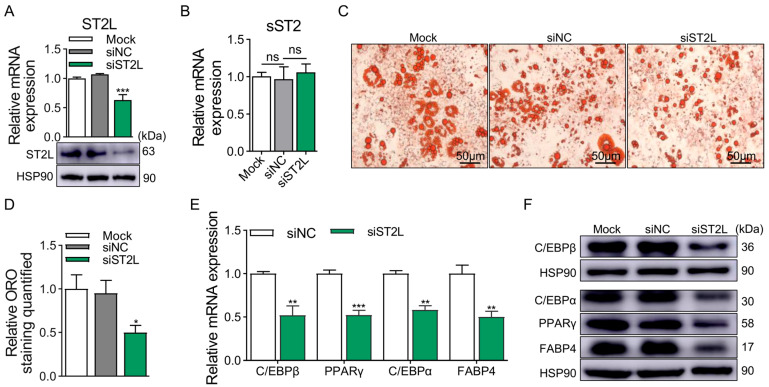
KD of ST2L reduces adipogenesis of 3T3-L1 cells. (**A**) KD efficiency of ST2L at mRNA (top panel) and protein (bottom panel) levels. (**B**) The mRNA levels of sST2 after KD of ST2L. (**C**) ORO staining of differentiated 3T3-L1 cells at D8. (**D**) Quantification of ORO staining. Scale bar, 50 μm. (**E**) The mRNA levels of adipogenic marker genes at D8, except for C/EBPβ at D2, were detected by qRT-PCR. (**F**) The protein levels of adipogenic marker genes at D8, except for C/EBPβ at D2, were detected by Western blotting. Data were represented as the mean ± SEM (*n* = 3). *, *p* < 0.05; **, *p* < 0.01; ***, *p* < 0.001.

Then, we focused on the impact of KD of ST2L on the adipogenesis of 3T3-L1 cells by transfecting specific siST2L. The mRNA and protein levels of ST2L demonstrated the efficiency of siST2L (Figure 6A). Further, we detected the mRNA expression of sST2 after the KD of ST2L to ensure that the specific siRNA targeting ST2L only downregulates the expression of ST2L and does not affect the expression of sST2. (Figure 6B) The results of ORO staining and quantitative analysis showed that the lipid droplet accumulation was significantly decreased in the ST2L KD group at D8 (Figure 6C,D). In addition, KD of ST2L significantly decreased the expression of adipogenic marker genes at both the mRNA and protein levels. (Figure 6E,F). According to the data above, it is concluded that the ST2 promotes adipogenesis of 3T3-L1 cells.

### 2.4. IL-33/ST2 Affects the Expression of TRAF6 and RelA in 3T3-L1 Cells

**Figure 7:**
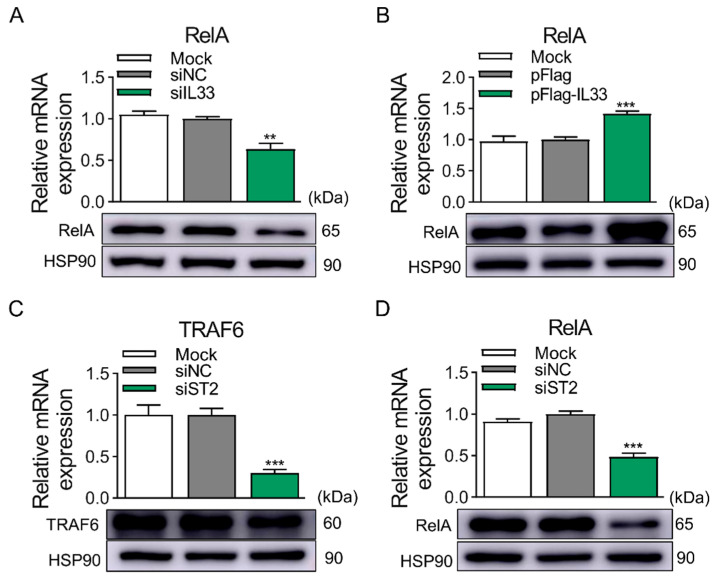
IL-33/ST2 axis affects the expression of TRAF6 and RelA. (**A**) The mRNA levels (top panel) and protein levels (bottom panel) of RelA after KD of IL-33. (**B**) The mRNA levels (top panel) and protein levels (bottom panel) of RelA after OE of IL-33. (**C**) The mRNA levels (top panel) and protein levels (bottom panel) of TRAF6 after KD of ST2. (**D**) The mRNA levels (top panel) and protein levels (bottom panel) of RelA after KD of ST2. Data were represented as the mean ± SEM (*n* = 3). **, *p* < 0.01; ***, *p* < 0.001. All cell samples used in the above results came from previous functional investigations.

Next, this study explored the potential regulatory mechanisms of the IL-33/ST2 axis in modulating the differentiation of 3T3-L1 preadipocytes, with TRAF6 and RelA serving as the subjects of investigation. Their expression profiles showed that the mRNA and protein expression levels exhibited a tendency of dynamic alterations during adipogenesis of 3T3-L1 preadipocytes (Figure S1). This suggested that TRAF6 and RelA may participate in the regulation of adipogenesis in 3T3-L1. Subsequently, this study investigated the relationship between IL-33 and the expression of RelA through OE and KD of IL-33. The results showed a positive correlation between the expression of IL-33 and that of RelA. Compared to the control group, the expression of RelA decreased due to the KD of IL-33, and OE of IL-33 led to a significant increase in the expression of RelA at both the mRNA and protein levels (Figure 7A,B). The results suggested a positive correlation between ST2 and TRAF6/RelA by KD of ST2. KD of ST2 significantly decreased the expression of TRAF6 and RelA at both the mRNA and protein levels compared to the control group (Figure 7C,D). All cell samples used in the KD group experiments in the above results came from previous functional investigations. This result suggested that IL-33/ST2 may regulate adipogenesis through regulating the expression of TRAF6/RelA.

### 2.5. TRAF6 Regulates the Expression of RelA to Promote Adipogenesis of 3T3-L1 Cells

**Figure 8:**
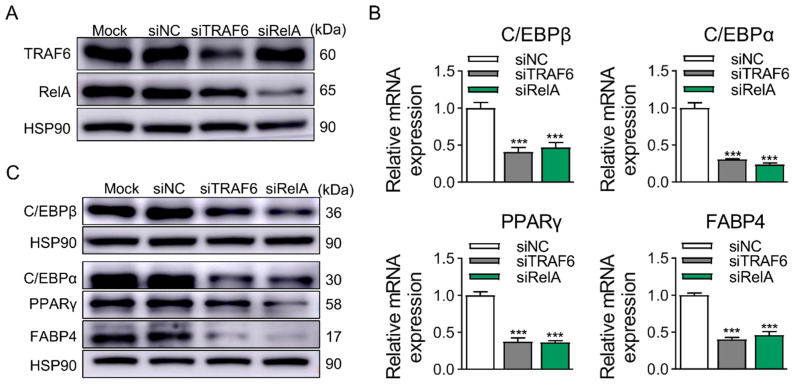
TRAF6 regulates the expression of RelA to promote adipogenesis of 3T3-L1 cells. (**A**) The regulatory relationship between TRAF6 and RelA was detected by Western blotting. (**B**) KD of TRAF6 or RelA, mRNA levels of adipogenic marker gene at D8, except for C/EBPβ at D2, were detected by qRT-PCR. (**C**) KD of TRAF6 or RelA, protein levels of adipogenic marker gene at D8, except for C/EBPβ at D2, were detected by Western blotting. Data were represented as the mean ± SEM (*n* = 3). ***, *p* < 0.001.

The regulatory relationship between TRAF6 and RelA was investigated by transfecting specific siRNA targeting TRAF6 or RelA. The results indicated that, compared with the control group, the downregulation of TRAF6 expression causes a decline in RelA expression, while the reduction of RelA has no impact on the expression of TRAF6 (Figure 8A). Moreover, the KD of TRAF6 or RelA downregulated the mRNA and protein expression levels of adipogenic marker genes of 3T3-L1 cells at D8 (Figure 8B,C). It can be inferred that TRAF6 is located upstream of RelA, and the TRAF6/RelA pathway is positively correlated with adipogenesis in 3T3-L1 cells.

## 3. Discussion

Functional research on cellular signaling pathways has always been a hot topic, including proliferation and differentiation. The objective of this research is to investigate the regulatory mechanism of the IL-33/ST2 axis on the differentiation of 3T3-L1 preadipocytes, since IL-33/ST2 is a recently discovered significant signaling pathway that governs the beige transformation of white adipose tissue and the thermogenesis of beige adipose tissue [27,28,29]. This study demonstrates that the IL-33/ST2 axis affects the adipogenesis of 3T3-L1 preadipocytes by regulating the expression of the TRAF6/RelA pathway. Therefore, the mechanism of the IL-33/ST2 axis that affects adipogenesis through regulating the TRAF6/RelA pathway provides novel ideas and perspectives for improving the molecular mechanism of adipogenesis. In addition, it may also provide an effective target for the treatment of obesity and other related diseases.

The IL-33/ST2 axis is involved in various biological regulatory processes such as inflammatory response [16], cell proliferation and migration [14,30], cell differentiation [15,31], and hormone regulation [32]. Lack of IL-33 or ST2 leads to an absence of UCP1 proteins, which impairs uncoupled respiration and thermoregulation [27]. Some adipokines can regulate adipocyte differentiation through paracrine or autocrine pathways by proteomic methods, such as IL-33 and ST2 [33]. The IL-33/ST2 axis participates in the modulation of the expression of multiple signaling pathways [13]. However, the exact mechanism by which the IL-33/ST2 axis governs the expression of these pathways and affects preadipocyte differentiation regulatory mechanisms remains not fully understood. Recently, the study investigated the process through which IL-33 regulates adipogenesis in preadipocytes through the Wnt/β-catenin/PPAR-γ signaling pathway [31]. In this study, we explored the mechanism by which the IL-33/ST2 axis affects adipogenesis by regulating the TRAF6/RelA pathway.

Firstly, we detected that the expression profiles of IL-33, sST2, and ST2L demonstrated alterations along with the advancement of induced differentiation, indicating that the IL-33/ST2 axis might be implicated in the modulation of 3T3-L1 preadipocyte differentiation. IL-33 has been confirmed to be a “dual-functional” cytokine, capable of regulating gene transcription and being secreted extracellularly as a ligand to bind with ST2L, transmitting extracellular signals to the interior and exerting biological effects [20,34]. According to the outcomes, IL-33 is inversely correlated with adipogenesis in 3T3-L1 preadipocytes. KD of endogenous IL-33 significantly promoted lipid droplet accumulation and the expression of adipogenic marker genes in 3T3-L1 cells, while OE of IL-33 showed the opposite effect.

Previous studies have explored the effect of the IL-33/ST2 axis on adipogenesis by upregulating/downregulating the expression of IL-33 [10,15,17,31,35]. However, the specific mechanism of action of IL-33 remains unclear. Different studies hold diverse opinions on the secretion form of IL-33 [20,21,36,37]. Whether IL-33 is secreted extracellularly in the cleaved mature form of IL-33 or the full-length form to participate in the regulation of the IL-33/ST2 axis requires further investigation [19,20,21,37]. However, it is indisputable that ST2L, as the receptor of IL-33, participates in the regulation of the IL-33/ST2 axis [16,21,38]. Therefore, this study investigates the function of ST2 in adipogenesis to explore the function and potential regulatory mechanism of the IL-33/ST2 axis on adipogenesis. ST2 is primarily composed of transmembrane ST2L and sST2, with sST2 competitively binding to IL-33 [16,21]. IL-33 was found to attenuate endoplasmic reticulum stress and apoptosis in glomerular endothelial cells, and sST2 treatment significantly reversed these effects of IL-33 [39]. Studies have revealed that sST2 attenuates the biological effects of IL-33 and aggravates various cardiovascular diseases when exploring potential novel therapeutic targets for cardiovascular diseases [40]. It has also been reported that sST2 plays a similar role in exploring the effects of the IL-33/ST2 axis on vascular and metabolic processes. IL-33 might play a crucial role in tissue preservation and repair in response to injury; nonetheless, the actions of IL-33 are mitigated by sST2 [41]. It can be seen that sST2 has an inhibitory effect on the function of IL-33.

Our research results indicate that KD the expression of ST2 significantly attenuates the accumulation of lipid droplets and the expression of adipogenic marker genes when 3T3-L1 cells are transfected with siRNA designed for the common coding region of the ST2 gene; OE of sST2 significantly augments lipid droplet accumulation and the expression of adipogenic marker genes in 3T3-L1 cells. The foregoing results imply that sST2 exhibits a positive correlation with adipogenesis in 3T3-L1 preadipocytes. To further explore the function of ST2, we designed siRNA in a distinctive region at the 3′ end of the coding region of ST2L, which will merely KD the expression of ST2L without influencing the expression of sST2, thereby conducting further study on the impact of ST2 on the differentiation of 3T3-L1 cells. The results suggest that ST2 promotes adipogenesis in 3T3-L1 preadipocytes, encompassing both ST2L and sST2.

From the above results, it can be seen that the functions of IL-33 and ST2 on adipogenesis are not consistent, and the factors contributing to this outcome are multifaceted. The full-length IL-33 has been validated to be able to transfer to the nucleus and bind to heterochromatin, acting as a “nuclear repressor” that may affect the activity of transcription factors [17,18,20]. NF-κB is a classic inflammatory signaling pathway, so in subsequent experiments, we further investigated the relationship between IL-33 and the expression level of transcription factor NF-κB. Additionally, it can be secreted into the extracellular matrix when cells are stimulated or injured to bind with the heterodimeric receptor complex consisting of ST2L and IL-1RAcP, transmitting extracellular signals intracellularly to exert an “alarm” function [18,34]. The form and secretion mode by which IL-33 is secreted extracellularly and binds to ST2 receptors to participate in the IL-33/ST2 axis remains controversial. However, it is certain that nuclear IL-33 acts as a “nuclear repressor” to inhibit the transcription of target genes [17,18,20]. Therefore, this study demonstrated the inhibitory effect of biologically active full-length IL-33 on adipogenesis. We speculate that this might be one of the reasons for the inconsistent functions of IL-33 and ST2. Our findings about the function of IL-33 on adipogenesis are moderately consistent with previous studies, which demonstrate that IL-33 inhibits adipogenesis in preadipocytes [15,31]. The inhibitory effect of IL-33 on preadipocyte differentiation across various species has been reported, further validating the credibility of our findings [10,15,31,42]. We validate that sST2 promotes adipogenesis during the differentiation of 3T3-L1 preadipocytes, which is in accordance with previous studies reporting that sST2 inhibits the function of IL-33 [16,21,39,40,41]. Furthermore, we also investigated the function of ST2L in the differentiation of 3T3-L1 preadipocytes, specifically by KD of the expression of ST2L. The experimental outcomes indicated that KD of the expression of ST2L attenuated the adipogenesis of 3T3-L1 preadipocytes. Therefore, we confirm that IL-33 suppresses the adipogenesis of 3T3-L1 preadipocyte, while ST2 promotes it. We investigated the signaling pathways involved in the IL-33/ST2 axis and used this as an entry point to explore the potential regulatory mechanisms by which the IL-33/ST2 axis regulates the differentiation of preadipocytes.

The IL-33/ST2 axis participates in the regulatory modulation of the expression of multiple signaling pathways, such as MAPKs, PI3K, AKT, mTOR, PLD, and NF-κB pathways associated with adipocyte differentiation [13,17]. Previous studies have indicated that the IL-33/ST2 axis is capable of activating the NF-κB signaling pathway, but the underlying regulatory mechanism remains ambiguous [43,44]. TRAF6 forms a complex with the E2 ubiquitin-conjugating enzymes and connects the K63-linked ubiquitin chain to the lysine residues on various target proteins, including itself and IKKγ/NEMO [22,24,25]. These ubiquitin chains form complexes that activate kinases through linkers containing ubiquitin-binding domains, such as TAB2/3. TAK1/3 recruits kinase TAK1, which activates the IKK kinase complex (IKKβ and IKKα), leading to the activation of MAPK and NF-κB transcription factors [26,45,46]. Moreover, among the NF-κB/Rel family (RelA, RelB, c-Rel, p100/p52, and p105/p50), except for RelA, whose promoter region contains no NF-κB response element and thus is not autoregulated by the NF-κB pathway, the rest are all autoregulated by the NF-κB pathway [47]. Therefore, we focused on the IL-33/ST2/TRAF6/RelA axis to further investigate the potential mechanism of the IL-33/ST2 axis in regulating preadipocyte differentiation. The results demonstrated that the expression levels of TRAF6 and RelA presented dynamic alterations, along with the advancement of induced differentiation, indicating that TRAF6 and RelA may also be involved in the adipogenesis process of preadipocytes. Moreover, the expression of TRAF6 decreased due to KD of ST2. The expression of RelA diminished due to the KD of IL-33 or ST2, while its expression increased due to the OE of IL-33. Summarizing the above results, the full-length IL-33 and ST2 have inconsistent functions in adipogenesis, but downregulation of IL-33 and ST2 expression inhibits the expression of RelA. Previous studies have demonstrated that IL-33 acts as a negative regulator of NF-κB, providing a plausible explanation and evidence for the inconsistent functions of IL-33 and ST2 in the adipogenesis of 3T3-L1 preadipocytes observed in this study, despite their consistent effects on the expression level of RelA [20,43]. We believe that the different mechanisms by which full-length IL-33 and ST2 regulate the expression of RelA lead to this result. Knocking down ST2 inhibits IL-33/ST2 axis signaling and suppresses the activation of IKK kinase complexes (IKK β and IKK α), thereby suppressing the expression of RelA. Knocking down IL-33 inhibits the expression of RelA because full-length IL-33 is mainly localized in the nucleus due to its interaction with RelA. This interaction occurs between the N-terminal part of IL-33 from aa 66–109 and the N-terminal Rel homology domain of RelA. The formation of the IL-33/NF-κB complex results in decreased binding of RelA to its cognate DNA and impaired p65-mediated transactivation [17,20,48]. We believe that this is the main reason for the inconsistent functions of IL-33 and ST2 in adipogenesis, despite their positive correlation with RelA expression. Our results further demonstrate that the OE of IL-33 impacts more RelA to attenuate adipogenesis. Overall, the IL-33/ST2 axis regulates the expression of TRAF6/RelA, and the IL-33/ST2 axis is positively correlated with the expression of TRAF6/RelA.

PRDX1’s inhibition of TRAF6 activity brings about the inhibition of NF-κB activation and autophagy activation [49]. The miR-146a-5p/TRAF6/NF-κB p65 axis drives pancreatic chemoresistance by regulating P-glycoprotein [50]. Overexpression of TRAF6 promoted the expression of p-NF-κB p65 and inhibited the Gastrodin-induced effects [51]. miR-146a enhances the survival of intestine epithelial cells under ischemia and I/R injury via TRAF6 inhibition, causing a reduction in NF-κB p65 nuclear translocation [52]. There are also studies investigating the function of TRAF6 or RelA in adipogenesis of preadipocytes among various species, which offer us some inspiration [53,54,55]. Therefore, the regulatory relationship between TRAF6 and RelA was investigated, as well as their function on adipogenesis in 3T3-L1 cells. According to the results, TRAF6 is upstream of RelA as the downregulation of TRAF6 expression causes a decline in RelA expression, while the reduction of RelA has no impact on the expression of TRAF6, and both TRAF6 and RelA accelerate adipogenesis in preadipocytes. Consequently, TRAF6 affects adipogenesis in 3T3-L1 preadipocytes by regulating the expression of RelA.

In conclusion, we confirm that IL-33 is negatively associated with adipogenesis in 3T3-L1 preadipocytes, while ST2 is positively associated with it, encompassing both ST2L and sST2. Furthermore, IL-33/ST2 affects adipogenesis in 3T3-L1 preadipocytes through regulating the TRAF6/RelA pathway. Taken together, this study will provide new ideas and insights for improving the molecular mechanism of adipogenesis and may also serve as a novel therapeutic target for obese patients.

## 4. Materials and Methods

### 4.1. Cell Culture and Induced Adipogenic Differentiation

Mouse preadipocytes 3T3-L1 were purchased from Procell Life Science & Technology Company Limited (Wuhan, China). Cells were cultured in DMEM medium containing 10% fetal bovine serum and 1% penicillin-streptomycin in an incubator at 37 °C with 5% CO_2_. The detailed induction protocol was as follows: Cells were induced by 3-isobutyl-1-methylxanthine (IBMX), dexamethasone (Dex), and insulin (MDI) induction medium containing 1 μM Rosiglitazone for 2 days following 2 days of cell contact inhibition. Then, the cells were treated with an insulin induction medium for another 2 days. Finally, the cells were treated with a complete medium for 4 days. Ninety percent of cells with obvious lipid droplets are considered mature adipocytes.

### 4.2. Vector Construction

CDS of IL-33 or sST2 were ligated to the multiple cloning sites of the pCMV vector by seamless cloning techniques, after the pCMV vector with the Flag Tag at the N-terminal was cleaved by *Xho*I and *Not*I, respectively. The constructed recombinant vectors were named pFlag-IL-33 or pFlag-sST2, respectively. Vector construction primers are listed in Table S1.

### 4.3. Cell Transfection

Cells were transfected with Lipo8000^TM^ transfection reagent (Beyotime, Shanghai, China) at approximately 80% confluence. The cells were proportionately inoculated into the 6-well plates one day before transfection to ensure that the cell density reached 80–90% around 16 h. The DMEM complete medium of well plates was replaced with fresh DMEM medium without serum and antibiotics before transfection. The protocol of transfection is as follows: (1) For the pCMV vector, 3 µg of plasmid per well was transfected in 6-well plates. (2) For siRNAs, cells were transfected with 60 nM/well. siRNAs were synthesized in Shanghai GenePharma Co., Ltd. (Shanghai, China), as listed in Table S2. The transfection efficiency of mRNA or protein was analyzed at 24 h or 48 h after transfection, respectively.

### 4.4. Synthesis of cDNA and Quantitative Real-Time PCR

The total RNA of cells was isolated by the RNeasy™ Animal RNA Isolation Kit with Spin Column (Beyotime, Shanghai, China). cDNAs of total RNA were then synthesized using the BeyoRT™ First Strand cDNA Synthesis Kit (RNase H minus). The reaction components of quantitative real-time PCR (qRT-PCR) were prepared by 2× Universal SYBR Green Fast qRT-PCR Mix (ABclonal, Wuhan, China) according to the guidelines in the kit’s manual. The obtained data were analyzed by the 2^−ΔΔCT^ formula. Mouse β-actin in 3T3-L1 cells was used as internal reference genes for mRNAs. The primers used for qRT-PCR analysis are listed in Table S3.

### 4.5. Western Blotting

Cells were lysed using radioimmunoprecipitation assay lysis buffer plus 1% protease inhibitors (Beyotime, Shanghai, China). The supernatant solution containing total protein was collected by centrifugation. Protein concentration was measured using the Enhanced BCA Protein Kit (Beyotime, Shanghai, China). Total proteins separated by SDS-PAGE were blotted onto the PVDF membrane. Next, blocked PVDF membranes were incubated with primary antibodies, followed by HRP-labeled secondary antibodies. After chemiluminescence reactions by the BeyoECL Plus Kit, membranes were visualized and photographed using Amersham Imager 600 (GE, Piscataway, NJ, USA). The antibody information used is listed in Table S4.

### 4.6. Oil Red O (ORO) Staining and Quantitative Analysis

According to the protocol given by the manufacturer, the working liquid is prepared by a 5:2 ratio of ORO storage liquid and diluent. Before staining 3T3-L1 cells, slowly wash with PBS preheated at 37 °C 3–5 times to clean the remaining medium. The dye on the cell surface was washed away using 60% isopropyl alcohol after the cells had been impregnated for 12–15 min. According to the experimental requirements, choose whether to re-stain with hematoxylin dye solution for 3–5 min, then wash with PBS. The stained lipid droplets inside the cells are observed through an inverted microscope (Zeiss, Jena, Germany). After the cells are soaked in 100% isopropyl alcohol for 3–5 min, gently moisten the liquid and collect. Drip into the 96-well plate to detect the accumulation of lipid droplets by measuring the optical density using a microplate detector (Biotek, Winooski, VT, USA) at 480 nm.

### 4.7. Statistical Analysis

All data obtained were analyzed by GraphPad Prism software (Ver. 8.0), and the data represent the mean  ±  SEM (*n* = 3). Statistical analysis of significant differences between the two groups or multigroup comparisons were performed by Student’s *t*-test or one-way ANOVA/Tukey’s test, respectively (*, *p* < 0.05; **, *p* < 0.01; ***, *p* < 0.001).

## Data Availability

The data presented in this study are available on request from the corresponding author.

## Supplementary Materials

The following supporting information can be downloaded at: https://www.mdpi.com/article/10.3390/ijms252212005/s1.
