# Supplementary material for: The IL-33/ST2 Axis Affects Adipogenesis Through Regulating the TRAF6/RelA Pathway
Source: Int J Mol Sci. 2024 Nov 8;25(22):12005. doi: 10.3390/ijms252212005 (PMC11593896; doi:10.3390/ijms252212005)
Supplement: Supplementary file 1 [file ijms-25-12005-s001.zip › ijms-3224602-supplementary.pdf]

Supplementary Data

The *IL-33*/*ST2* Axis Affects Adipogenesis through Regulating the *TRAF6*/*RelA* Pathway

Shujun Cao[1†], Xuyong Qin[1†], Chengping Li[1], Lichun Zhang[2], Shizhong Ren[1], Wenhao Zhou[1], Meiman Zhao[1] and Guoli Zhou[1*]

AUTHOR INFORMATION

*** Corresponding Author**

Guoli Zhou- College of Agriculture and Biology, Liaocheng University, Liaocheng 252000, China;

Email: glzhou1975@163.com; Phone: +86- 635-8230050

† These authors contributed equally to this work.

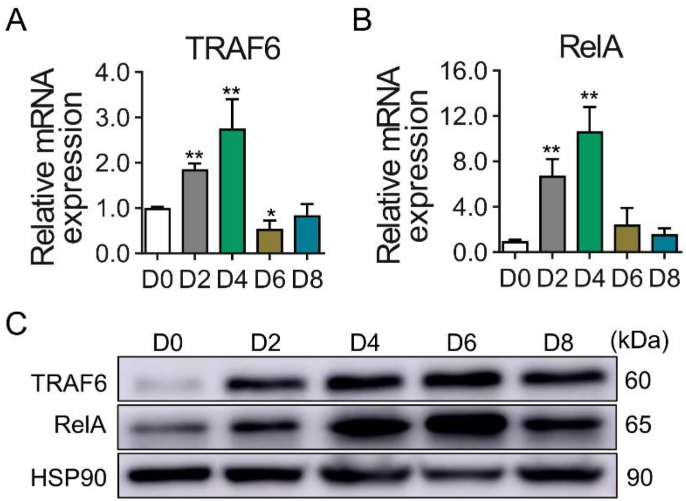

Figure S1. Expression Profile of TRAF6 and RelA During Adipogenesis.
(A) and (B) The mRNA levels of TRAF6 and RelA were detected by qRT-PCR.
(C) Protein levels of TRAF6 and RelA were detected by Western blotting. Data were represented as the mean ± SEM (*n* = 3). Data were represented as the mean ± SEM (*n* = 3). *, *P* < 0.05; **, *P* < 0.01.
With the increase of induced differentiation process, the mRNA and protein expression levels of TRAF6 and RelA exhibited a tendency of initial increase followed by decrease.

Table S1. Primers used for the construction of recombinant vectors

Name of constructs	Sequence of primer (5′→3′)
pFlag-IL-33	F: GTCGACCGAGATCTCTCGAGGTATGAGACCTAGAATGAAGTATTCCA
	R: TGTCTGGATCCCCGCGGCCGCTTAGATTTTCGAGAGCTTAAACATA
pFlag-sST2	F: CGACAAGCT TATGCGAATTCGGATGATTGACAGACAGAGAATGGGA
	R: TGTCTGGATCCCCGCGGCCGCTCAAGCAATGTGTGAGGGACAC

Table S2. List of siRNAs

Name	Sense strand (5′→3′)	Antisence strand (5′→3′)
siNC	S: UUCUCCGAACGUGUCACGUTT	A: ACGUGACACGUUCGGAGAATT
siIL-33	S: GCAUCCAAGGAACUUCACUTT	A: AGUGAAGUUCCUUGGAUGCTT
siST2	S: GCUGCAAUAUCCCUGAUUATT	A: UAAUCAGGGAUAUUGCAGCTT
siST2L	S: GGAUGUAGUUUAUUGCUAATT	A: UUAGCAAUAAACUACAUCCTT
siTRAF6	S: GAGAACAGAUGCCUAAUCATT	A: UGAUUAGGCAUCUGUUCUCTT
siRelA	S: GAUCAAUGGCUACACAGGATT	A: UCCUGUGUAGCCAUUGAUCTT

Table S3. Primers used for qRT-PCR

Name	GenBank ID	Forward primer (5′→3′)	Reverse primer (5′→3′)
IL-33	NM_001164724.2	F: ATTTCCCCGGCAAAGTTCAG	R: AACGGAGTCTCATGCAGTAGA
sST2	NM_001294171.2	F: GGCTGATGTCCTGTGGCAG	R: TCAAGCAATGTGTGTGAGGGAC
ST2L	NM_001025602.4	F: GAACGATGGCAAGCTCTACG	R: GCCCAGGTAACAGGTCTCT
ST2	NM_001025602.4	F: AGAGAATGGGACTTTGGGCTT	R: GGCTGGTAGAAACTTCAGACG
TRAF6	NM_001303273.1	F: AAAGCGAGAGATTCTTTCCCTG	R: ACTGGGGACAATTCACTAGAGC
RelA	NM_009045.5	F: TCCTGTTCGAGTCTCCATGCAG	R: GGTCTCATAGGTCCTTTTGCGC
C/EBPα	NM_001287514.1	F: CTGATTCTTGCCAAACTGAG	R: GAGGAAGCTAAGACCCACTAC
C/EBPβ	NM_001287738.1	F: GCAAGAGCCGCGACAAGG	R: GGCTCGGGCAGCTGCTT
PPARγ	NM_001127330.3	F: TCCGTAGAAGCCGTGCAAGAGATCA	R: CAGCAGGTTGTCTTGGATGTCCTCG
FABP4	NM_024406.4	F: TGAAAGAAGTGGGAGTGGGCTTTGC	R:CACCACCAGCTTGTCACCATCTCGT
mβ-actin	NM_007393.5	F: CCAGCCTTCCTTCTTGGGTATGG	R: ACGCAGCTCAGTAACAGTCCG

Table S4. Antibodies used for Western Blotting

Name	Catalogue Number	Source of antibodies
anti-C/EBPβ	AF2134	Beyotime, Shanghai, China
anti-C/EBPα	A0904	ABclonal, Wuhan, China
anti-PPARγ	A11183	ABclonal, Wuhan, China
anti-FABP4	A6843	Beyotime, Shanghai, China
anti-FABP4	A0232	ABclonal, Wuhan, China
anti-IL-33	A8096	ABclonal, Wuhan, China
anti-IL-33	AF7275	Beyotime, Shanghai, China
anti-ST2	K106835P	Solarbio, Beijing,China
anti-TRAF6	A23385	ABclonal, Wuhan, China
anti-NF-κB p65/RelA	A19653	ABclonal, Wuhan, China
anti-HSP90	A5006	ABclonal, Wuhan, China
anti-Flag Tag	AE092	ABclonal, Wuhan, China
HRP-labeled Goat Anti-Rabbit IgG(H+L)	AS014	ABclonal, Wuhan, China
